# Immobilization Decreases FOXO3a Phosphorylation and Increases Autophagy-Related Gene and Protein Expression in Human Skeletal Muscle

**DOI:** 10.3389/fphys.2019.00736

**Published:** 2019-06-14

**Authors:** Andreas Buch Møller, Mikkel Holm Vendelbo, Peter Schjerling, Christian Couppé, Niels Møller, Michael Kjær, Mette Hansen, Niels Jessen

**Affiliations:** ^1^Research Laboratory for Biochemical Pathology, Institute for Clinical Medicine, Aarhus University, Aarhus, Denmark; ^2^Steno Diabetes Center Aarhus, Aarhus University Hospital, Aarhus, Denmark; ^3^Department of Nuclear Medicine & PET-Centre, Aarhus University Hospital, Aarhus, Denmark; ^4^Department of Orthopaedic Surgery M, Bispebjerg Hospital, and Center for Healthy Aging, Faculty of Health and Medical Sciences, Institute of Sports Medicine, University of Copenhagen, Copenhagen, Denmark; ^5^Musculoskeletal Rehabilitation Research Unit, Department of Physical Therapy, Bispebjerg Hospital, Copenhagen, Denmark; ^6^Medical Research Laboratory, Institute for Clinical Medicine, Aarhus University, Aarhus, Denmark; ^7^Section of Sports Science, Department of Public Health, Aarhus University, Aarhus, Denmark

**Keywords:** autophagy, immobilization, skeletal muscle, human, FOXO3a, autophagy-related genes

## Abstract

Immobilization of the lower limbs promotes a catabolic state that reduces muscle mass, whereas physical training promotes an anabolic state that increases muscle mass. Understanding the molecular mechanisms underlying this is of clinical interest, as loss of muscle mass is a major complication to critical illness in humans. To determine the molecular regulation of protein synthesis and degradation during muscle loss and hypertrophy, we examined skeletal muscle biopsies from healthy human subjects after 2 weeks unilateral immobilization of a lower limb and during 6 weeks of physical rehabilitation. We have previously shown that cross-sectional area of the knee muscle-extensors decreased by ∼10% during immobilization and was completely restored during rehabilitation. Here we provide novel data to suggest that autophagy is an important underlying mechanism involved in regulation of muscle mass. Protein expression of MuRF1 and ATROGIN-1 did not change during the study, indicating that the recruitment of substrates to the proteasomes was unaltered. Phosphorylation of mTORat Ser^2448^ did not change during the study, and neither did phosphorylation of the mTORC1 substrates 4EBP1 Thr^37/46^ and p70S6K Thr^389^, suggesting that this pathway does not suppress protein synthesis during muscle wasting. Protein levels of p62 and ULK1 increased during immobilization and returned to baseline levels during rehabilitation. Same pattern was observed for FOXO3a phosphorylation at Ser^318/321^, suggesting transcriptional activation during immobilization and inactivation during rehabilitation. To investigate this further, we analyzed mRNA expression of seven autophagy-related genes controlled by FOXO3a. Five of these (p62, LC3B, BECLIN-1, ATG12, and BNIP3) increased during immobilization and returned to baseline during rehabilitation. In conclusion, immobilization of a lower limb increases autophagy-related gene and protein expression in human skeletal muscle in a pattern that mirrors FOXO3a phosphorylation. These findings could imply that FOXO3a dependent transcriptional regulation of autophagy is involved in the regulation of muscle mass in humans.

**Clinical Trial Registration:**

The study was approved by the Ethics Committee of Copenhagen (j.no. H-1-2010-016).

## Introduction

Skeletal muscle is essential for maintaining health due to its large contribution to whole body metabolism (Argiles et al., 2015). Regulation of muscle mass is reflected by the relative rates of protein synthesis and degradation. Protein synthesis occurs on the ribosomes and protein degradation occurs via two proteolytic systems: the ubiquitin-proteasome and the autophagy-lysosome (Sandri, 2008). The molecular regulation of these systems involves transcriptional and non-transcriptional mechanisms that directs protein turnover to either anabolic or catabolic states (Sakuma et al., 2015). Understanding the mechanisms that regulates protein balance may uncover future strategies to combat severe muscle wasting in critical illness.

Immobilization of the lower limbs promotes a catabolic state that reduces muscle mass (Akima et al., 1997; Bodine et al., 2001a; Brocca et al., 2012; Wall et al., 2014). Suppressed protein synthesis contributes to muscle wasting in both rodents and humans (de Boer et al., 2007; Greenhaff et al., 2008; Phillips et al., 2009). mTORC1 is a key regulator of protein synthesis (Zoncu et al., 2011). mTORC1 activates p70S6K through phosphorylation at Thr^389^ and suppresses 4EBP1 activity through phosphorylation at Thr^37/46^, which facilitates transcriptional initiation and elongation (Zoncu et al., 2011). Suppressed mTORC1 activity has been observed in rodent immobilization studies (Bodine et al., 2001b; Hornberger et al., 2001; Talbert et al., 2013), but this has not been verified in humans, although decreased protein synthesis has been observed using tracer techniques (de Boer et al., 2007; Greenhaff et al., 2008; Phillips et al., 2009; Rennie et al., 2010; Wall et al., 2014). Thus, the degree to which reduced mTORC1 signaling contributes to muscle wasting in humans remains unclear.

Increased proteasome activity contributes largely to muscle wasting in rodents (Rennie et al., 2010; Bodine and Baehr, 2014). Specific enzymes (E3 ligases) mark proteins for proteasomal degradation and MuRF1 and ATROGIN-1 have been identified as muscle-specific E3 ligases (Bodine and Baehr, 2014). Protein expression of MuRF1 and ATROGIN-1 are elevated during immobilization in rodents (Bodine et al., 2001a; Krawiec et al., 2005), and deletion of these proteins reduce muscle loss during catabolic conditions in mice (Bodine et al., 2001a). In humans, gene expression of MuRF1 and ATROGIN-1 increase during immobilization (Nedergaard et al., 2012; Suetta et al., 2012; Wall et al., 2014), but protein expression appear unaltered, or even decreased, despite considerable loss of muscle mass (Nedergaard et al., 2012; Lundell et al., 2018). Thus, the role of this system in regulation of muscle mass remains controversial and needs further investigation.

Autophagy delivers proteins and organelles to the lysosomes for degradation (Sandri, 2013). Studies in mice indicate that increased autophagy plays a major role in muscle wasting during fasting and immobilization (Zhao et al., 2007; Dobrowolny et al., 2008; Talbert et al., 2013), but the involvement in human muscle wasting is unknown. Studies in cultured cells have demonstrated that autophagy is regulated through transcriptional and non-transcriptional mechanisms (Mammucari et al., 2007; Zhao et al., 2007). ULK1 is the main enzyme responsible for non-transcriptional regulation of autophagy (Russell et al., 2013). AMPK activates ULK1 by phosphorylation at Ser^555^ and mTORC1 inhibits ULK1 by phosphorylation at Ser^757^ (Kim et al., 2011; Jessen et al., 2014; Moller et al., 2015). During autophagy, LC3BI and GABARAPI are converted to their lapidated forms (LC3BII and GABARAPII) and incorporated in the autophagosomes where they functions as docking sites for adapter proteins, such as p62 and BNIP3 (Hanna et al., 2012; Sahani et al., 2014). FOXO3a plays an important role in transcriptional regulation of autophagy-related genes, including BECLIN-1, ATG12, ULK1, BNIP3, GABARAP, and LC3B (Mammucari et al., 2007; Zhao et al., 2007; O’Neill et al., 2016). Transcriptional activity of FOXO3a is regulated by phosphorylation: increased phosphorylation at Ser^318/321^ leads to transcriptional inactivation and dephosphorylation leads to activation (Tong et al., 2009). Foxo transcription factors have been ascribed a critical role in muscle wasting, as muscle-specific deletion of Foxo members protects from fasting-induced muscle loss in mice (Milan et al., 2015). These findings show that autophagy plays a prominent role in regulation of muscle mass and indicate that treatment strategies against muscle wasting should be targeted this system. However, there is a translational gap between studies in animals and humans that needs to be filled before such strategies can emerge. This gap is of particular importance when it comes to autophagy as important differences have been documented between rodents and humans (Fritzen et al., 2015).

The aim of the present study was to investigate molecular mechanisms involved in regulation of muscle mass during immobilization and rehabilitation in humans, with specific focus upon regulation of autophagy. Our hypothesis was that autophagy-related gene and protein expression are upregulated during immobilization and that these alterations are reversed when muscle mass is restored by physical rehabilitation.

## Materials and Methods

### Subjects

Seventeen postmenopausal females participated in the study. Functional and morphological data from these subjects have previously been published in a separate paper along with a detailed description of the trial protocol (Larsen et al., 2018). Prior to inclusion, the subjects underwent a screening interview aimed to assess their medical history and to estimate their maximal rate of oxygen consumption (VO_2_-max) by a two-step submaximal bike test. None of the subjects had a previous record of acute or chronic illness or took any medication affecting skeletal muscle mass or function. The subjects were informed about the purpose and risks related to the study and gave written informed consent to participate. The study was approved by the Ethics Committee of Copenhagen (j.no.H-1-2010-016), and performed in accordance to the Declaration of Helsinki.

### Study Design

**FIGURE 1:**
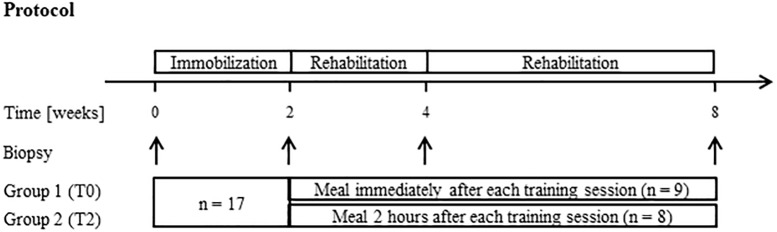
Schematic presentation of the study design. All subjects (*n* = 17) completed 2 weeks unilateral immobilization of a lower limb. The subjects were then randomized into two groups before completing 6 weeks rehabilitation consisting of conventional resistance training. Group 1 (T0, *n* = 9) consumed a high-protein meal immediately after each training session and Group 2 (T2, *n* = 8) consumed a similar meal 2 h after each training session. Skeletal muscle biopsies were sampled before and after immobilization, as well as after 2 and 6 weeks of rehabilitation.

All subjects underwent 2 weeks unilateral immobilization of the lower limb. The subjects were then randomized into two groups before completing 6 weeks rehabilitation consisting of three training sessions a week. One group consumed a high-protein meal immediately after each training session, whereas the other group consumed a similar meal 2 h after each training session. Skeletal muscle biopsies were sampled before and after immobilization, as well as after 2 and 6 weeks of rehabilitation. A schematic representation of the study is presented in Figure 1.

### Immobilization Protocol

Immobilization was performed by 2 weeks unilateral immobilization of a randomly selected lower limb using a Donjoy splint (DJO, CA, United States). The knee joint was fixed in 40 degrees to circumvent walking ability of the immobilized limb. The subjects were carefully instructed to perform all ambulatory activities on crutches and to abstain from ground contact.

### Rehabilitation Protocol

Rehabilitation consisted of 6 weeks machine based (Technogym, Gambettola, Italy) resistance training of the immobilized leg. The protocol aimed to induce skeletal muscle hypertrophy and included knee-extension and leg-press exercises. The protocol was progressively designed, stating with 3 sets of 12 repetitions at an intensity corresponding to 15 repetition maximum (RM) in the first week, and ending with 4 sets of 8 repetitions at an intensity of 10 RM in last week. The rest period between sets was 2 min. The training intensity was increased every week throughout the training period based on 5 RM tests performed at the beginning of the last weekly training session. Based on the 5 RM test, the intended workload was estimated and applied in the training. In addition to the prescribed resistance training, the subjects were allowed to walk (up to 10 km/day) or cycle (up to 25 km/day) or swim (up to 1 km/day), but not regularly (>2 times per week). No caloric intake was allowed 2 h up to each training session or 2 h after each training session except from the intervention meal.

### High-Protein Meals

Before entering the rehabilitation period, subjects were randomized into two groups. One group (T0) consumed high-protein meals immediately after each training session in the rehabilitation period, whereas the other group (T2) consumed a similar meal 2 h after each training session. Details regarding the meals have been published previously (Larsen et al., 2018).

### Skeletal Muscle Biopsy Sampling

Skeletal muscle biopsies were sampled after an overnight fast from vasus lateralis under local anesthesia using a Bergström needle and stored at -80°C until analyses were performed. The biopsies were sampled in the control leg prior to the immobilization (week 0) and in the immobilized leg immediately after 2 weeks immobilization (week 2), as well as after 2 and 6 weeks rehabilitation (week 4 and 8). Subjects were instructed not to participate in any exercise activities 48 h before biopsy sampling.

### Protein Extraction and Western Blot Analysis

Frozen muscle biopsies were freeze-dried 48 h in a Heto Drywinner (Heto Holten A/S, Allerød, Denmark) at -96°C and 0.5 mmHg. Freeze-dried muscle tissue were homogenized with ceramic beads in ice-cold lysis buffer (20 mM TRIS base, 50 mM NaCl, 250 mM sucrose, 1% (vol/vol) Triton-X100, 1% (vol/vol) HALT protease inhibitor cocktail (Thermo Fisher Scientific, MA, United States), 2 mM DTT, 5 mM nicotinamide, 10 mM Na_4_P_2_O_7_, 10 μl trichostatin A, 20 mM NaF, pH 7.4) using a Precellys 24 homogenizer (Bertin Technologies, FR). Insoluble materials were removed by centrifugation at 14,000 ×*g* for 20 min at 4°C. Protein concentration of the supernatant was determined using a Bradford assay (BioRad, CA, United States). Samples were adjusted to equal concentrations with milli-Q water and denatured by mixing with Laemmli’s buffer and heating at 95°C for 5 min. Equal amounts of protein were separated by SDS-PAGE using the BioRad Criterion system, and proteins were electroblotted onto PVDF membranes (BioRad). Control for equal loading was performed using the Stain-Free technology which has been shown to be superior to beta-action and GAPDH in human skeletal muscle (Gurtler et al., 2013; Vigelso et al., 2015). Membranes were blocked for 2 h in a 2% bovine serum albumin solution (Sigma-Aldrich, MO, United States) and incubated overnight with primary antibodies (antibodies are specified in Table 1). All antibodies have previously been used for western blot analysis of human muscle protein. Up to five proteins with different MW were detected at the same membrane. To do this, the molecular markers were used to cut the membranes into smaller shreds. Afterward, the shreds were incubated in different primary antibodies according the protein of interest. Different proteins with similar molecular weights were detected on separate membranes. After incubation in primary antibodies the membranes were incubated 1 h with HRP-conjugated secondary antibodies (antibodies are specified in Table 1). Proteins were visualized by chemiluminescence (Pierce Supersignal West Dura, Thermo Fisher Scientific, IL, United States) and quantified with ChemiDoc^TM^ MP imaging system (BioRad). Protein Plus Precision All Blue standards were used as markers of molecular weight (BioRad).

**Table 1:** Antibody and primer specifications.

Primary antibodies		
**Epitope**	**Manufacturer**	**Catalog no.**

ULK1	Cell Signaling	4773
p-ULK1 Ser^555^	Cell Signaling	5869
p-ULK1 Ser^757^	Cell Signaling	6888
mTOR	Cell Signaling	2972
mTOR Ser^2448^	Cell Signaling	2971
p62/SQSTM1	Abcam	ab56416
AKT-pan	Cell Signaling	3063
p-AKT Ser^473^	Cell Signaling	9271
p70S6K	Cell Signaling	9202
p-p70S6K Thr^389^	Cell Signaling	9205
LC3B	Cell Signaling	3868
ATG5	Cell Signaling	12994
GABARAP	Cell Signaling	13733
BECLIN-1	Cell Signaling	3738
FOXO3a	Cell Signaling	3839
p-FOXO3a Ser^318/321^	Cell Signaling	9465
4EBP1	Cell Signaling	9644
Non-p-4EBP1 Thr^37/46^	Cell Signaling	4923
MuRF1	Abcam	ab57865
ATROGIN-1	Abcam	Ab92281

**Secondary antibodies**		

**Epitope**	**Manufacturer**	**Catalog no.**

Rabbit Ig Mouse Ig	Santa Cruz Santa Cruz	Sc-2054 Sc-2096
Goat	Santa Cruz	Sc-2020
Streptavidin HRP	SouthernBiotech	7100-05

**Primers**		

**Target**	**GenBank**	**Primer**

ULK1	NM_003565.2	*Sense* GCCCTACACGCCATCTCCTCAA
		*Antisense* CCCGCATCTCAGCTCCCTGT
ATG12	NM_004707.3	*Sense* CCAGACCAAGAAGTTGGAACTCTCTAT
		*Antisense* GCAAGTTGATTTTCTTTGTGGTTC
GABARAPL1	NM_031412.2	*Sense* ACCTAGTGCCCTCTGACCTTACTGTTG
		*Antisense* CACTGGTGGGAGGGATGGTGTT
LC3B	NM_022818.4	*Sense* CAGATCCCTGCACCATGCCGT
		*Antisense* TTGGTTGGATGCTGCTCTCGAAT
p62/SQSTM1	NM_003900.4	*Sense* TGAAGAACGTTGGGGAGAGTGTG
		*Antisense* GGCTTCTTTTCCCTCCGTGCT
BNIP3	NM_004052.3	*Sense* GCATTGGAGAGAAAAACAGCTCACA
		*Antisense* GGGAATATTTTCCGGCCGACTT
BECLIN-1	NM_003766.3	*Sense* CGCAGCTGGATAAGCTGAAGAAAACC
		*Antisense* CGACCCAGCCTGAAGTTATTGATTG
MuRF1	NM_032588.3	*Sense* TGGGGGAGCCACCTTCCTCT
		*Antisense* ATGTTCTCAAAGCCCTGCTCTGTCT
RPLP0	NM_053275.3	*Sense* GGAAACTCTGCATTCTCGCTTCCT
		*Antisense* CCAGGACTCGTTTGTACCCGTTG
GAPDH	NM_002046.4	*Sense* CCTCCTGCACCACCAACTGCTT
		*Antisense* GAGGGGCCATCCACAGTCTTCT


*Specification of primary and secondary antibodies used for western blots, and primers used for RT-PCR.*

After initial problems with detecting LC3B lipidation using procedures as mentioned above, we extracted proteins from the remaining biopsy material using a lysis buffer containing stronger detergents [50 mM HEPES, 137 mM NaCl, 10 mM Na_4_P_2_O_7_, 20 mM NaF, 5 mM EDTA, 1 mM MgCl_2_, 1 mM CaCl_2_, 2 mM Na_3_VO_4_, 5 mM nicotinamide, 10 μM trichostatin A, 1% (vol/vol) HALT protease inhibitor cocktail (Thermo Fisher Scientific, MA, United States), 1% (vol/vol), NP-40, 10% (vol/vol) glycerol]. Biopsy material from week 0 and 2 existed from four subjects, which allowed us to examine LC3B lipidation before and after immobilization in four subjects.

### RNA Isolation and Real-Time RT-PCR Analysis

Frozen muscle biopsies (approximately 10 mg) were homogenized in 1 ml TriReagent using a FastPrep^®^-24 instrument (MP Biomedicals, Inc., Illkirch, France), with five steel beads and one siliciumcarbide crystal. Following homogenization (3 × 15 s), bromo-chloropropane was used to separate the samples into an organic phase and an aqueous phase. Total RNA was precipitated from the aqueous phase using isopropanol, and the pellet was washed in ethanol and subsequently dissolved in RNAse-free water. RNA concentrations were determined by spectroscopy at 260 nm and a good RNA integrity was ensured by gel electrophoresis. Five hundred nanogram of RNA was converted into cDNA in 20 μL using the OmniScript reverse transcriptase kit (Qiagen, CA, United States) and 1 μM poly-dT (Invitrogen) according to the manufacturer’s protocol (Qiagen). For each target mRNA, 0.25 μL cDNA was amplified in a 25 μL SYBR Green polymerase chain reaction containing 1 × Quantitect SYBR Master Mix (Qiagen) and 100 nM of each primer (Table 1). The amplification was monitored real time using a MX3005P Real-time PCR machine (Stratagene, CA, United States). The Ct values were related to a standard curve in order to determine the number of target mRNA molecules in each sample. The large ribosomal protein P0 (RPLP0) was chosen as internal control, as RPLP0 mRNA has been suggested to be constitutively expressed (Dheda et al., 2004). Comparison to another common reference mRNA, GAPDH, showed a transient decreased ratio of GAPDH normalized to RPLP0 after immobilization, indicating that one or the other was not stable. The change could be due to an increase in RPLP0 or a decrease in GAPDH. From a biological point of view it is more likely that GAPDH decreased (reduced glycolysis) than RPLP0 increased (higher protein synthesis) during an immobilization. Therefore, RPLP0 was chosen as a normalizer, but GAPDH shown in the figures. As can be seen from Figure 3, 5, the real targets generally change in the opposite direction of GAPDH, indicating that normalization with GAPDH would only have amplified the changes observed.

### Statistics

Subject characteristics were analyzed using Student’s *t*-test. The effects of immobilization/rehabilitation and timing of protein intake and their interactions were analyzed using a mixed-effect linear model with repeated measures for time. When a significant interaction or main effect was observed, linear comparison analysis, extracted from the mixed-effect linear model, was used to evaluate individual pairwise differences. Normal distribution and equal variance were assumed after graphical inspection of QQ-plots and Bland–Altman plots. Protein and mRNA data was log-transformed and presented as geometric means ± back-transformed SE. Data were analyzed in Stata (Stata 12.1, StataCorp LP, College Station, TX, United States), and graphs were designed in SigmaPlot (SigmaPlot 11.0, Sysstat Software, CA, United States).

## Results

### Skeletal Muscle Cross-Sectional Area (CSA) and Strength Decrease During Immobilization and Are Regained During Two Weeks of Physical Rehabilitation

Seventeen female subjects completed 2 weeks unilateral immobilization of a lower limb followed by 2 weeks physical rehabilitation. A schematic presentation of the protocol is included in Figure 1 and subject characteristics are presented in Table 2. We recently demonstrated that the included subjects lost ∼10% of their knee muscle-extensor CSA during 2 weeks immobilization and that this was associated with a 23% decrease in isometric muscle strength (Larsen et al., 2018). Moreover, CSA and isometric muscle strength was completely regained within 2 weeks rehabilitation (Larsen et al., 2018). During rehabilitation subjects consumed a high-protein meal immediately after (T0) or 2 h after (T2) each training session. Timing of protein intake did not affect changes in muscle mass and strength. The applied model is therefore ideal for studying molecular mechanisms involved in regulation of skeletal muscle mass during immobilization and rehabilitation in humans.

**Table 2:** Subject characteristics.

	All subjects	Group 1 (T0)	Group 2 (T2)
n	17	9	8
Age [years]	57 (50–70)	56 (51–64)	57 (50–70)
Weight [kg]	77.4 ± 2.9	77.4 ± 4.1	77.4 ± 4.4
Height [m]	1.65 ± 0.01	1.65 ± 0.01	1.66 ± 0.03
VO_2_-max [ml/kg/min]	25.3 ± 1.6	24.3 ± 2.1	26.5 ± 2.5


*Age is indicated as median and range. Weight, height, and VO_*2*_-max is indicated as mean ± SE.*

### mTORC1 Signaling Does Not Change During Immobilization and Physical Rehabilitation

**FIGURE 2:**
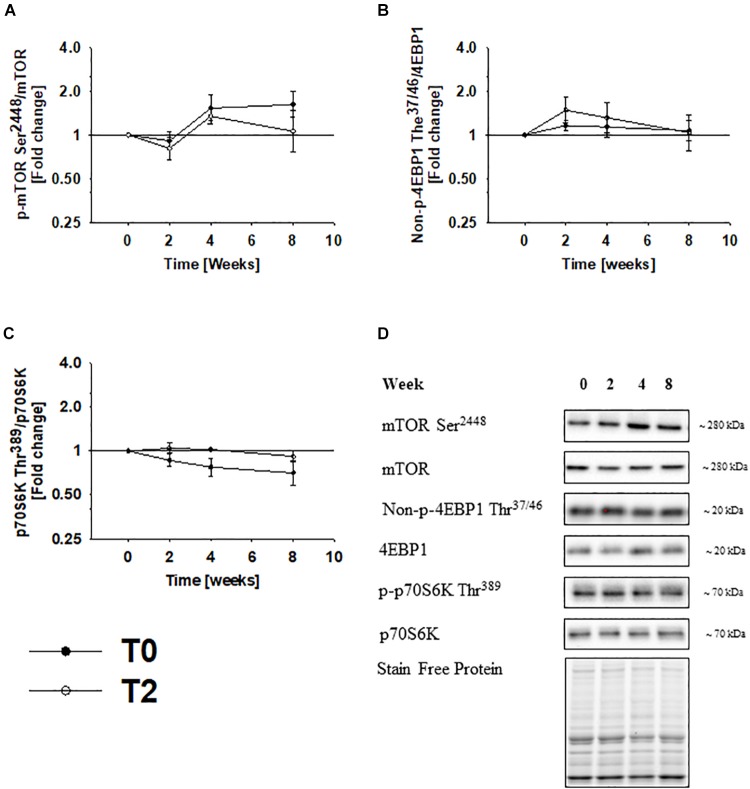
4EBP1 phosphorylation decreases during immobilization. mTOR phosphorylation at Ser^2448^ did not change during immobilization or rehabilitation **(A)**. In accordance, the mTORC1 substrates 4EBP1 phosphorylation Thr^37/46^ and p70S6K Thr^389^ remained unchanged during rehabilitation and rehabilitation **(B,C)**. Values are geometric means ± back transformed SE. Representative western blots **(D)**. Based on the applied molecular standards, approximated molecular weights are indicated on the right. Filled symbols represent subjects from T0 and open symbols represent subjects from T2.

To recapitulate previous findings from this study (Larsen et al., 2018), mTOR phosphorylation at Ser^2448^ did not change during immobilization or rehabilitation (Figure 2A). In accordance, phosphorylation of the mTORC1 substrates 4EBP1 Thr^37/46^ and p70S6K Thr^389^ remained unchanged during immobilization and rehabilitation (Figure 2B,C).

### Immobilization and Rehabilitation Does Not Change MuRF1 and ATROGIN-1 Protein Expression

**FIGURE 3:**
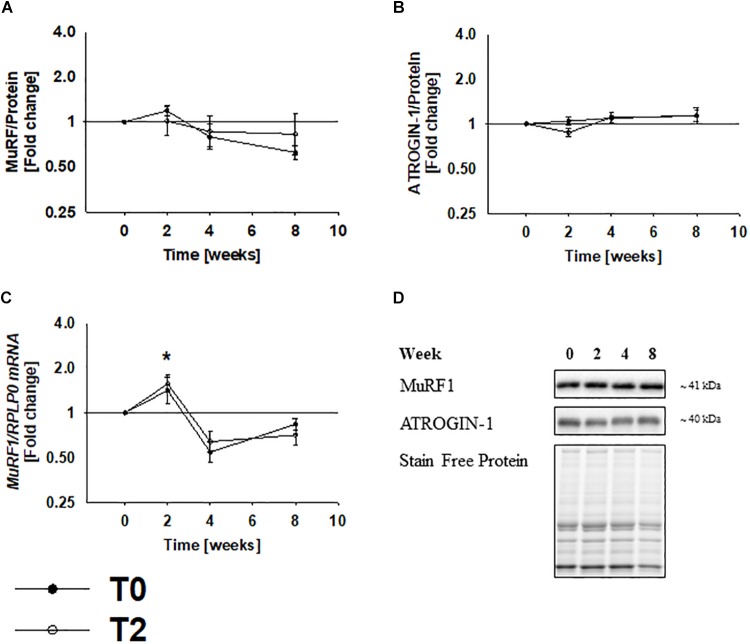
Protein expression of muscle-specific E3-ligases did not change during immobilization. MuRF1 and ATROGIN-1 protein expression did not change during immobilization and rehabilitation **(A,B)**. mRNA expression of MuRF1 increased during immobilization and decreased during the first 2 weeks of rehabilitation **(C)**. Values are geometric means ± back-transformed SE. ^∗^*Post hoc* test showed significant change during immobilization. Representative western blots **(D)**. Based on the applied molecular standards, approximated molecular weights are indicated on the right. Filled symbols represent subjects from T0 and open symbols represent subjects from T2.

To examine whether changes in muscle mass is associated with changes in markers of proteasomal degradation, we examined protein expression of two muscle-specific E3 ligases. As presented previously (Larsen et al., 2018), MuRF1 and ATROGIN-1 did not change during immobilization, which suggests that recruitment of proteins for proteasomal degradation is unchanged (Figure 3A,B). However, mRNA expression of MuRF1 increased by ∼60% during immobilization (Figure 3C).

### LC3B mRNA and p62 Protein Expression Increase During Immobilization and Normalize During Rehabilitation

**FIGURE 4:**
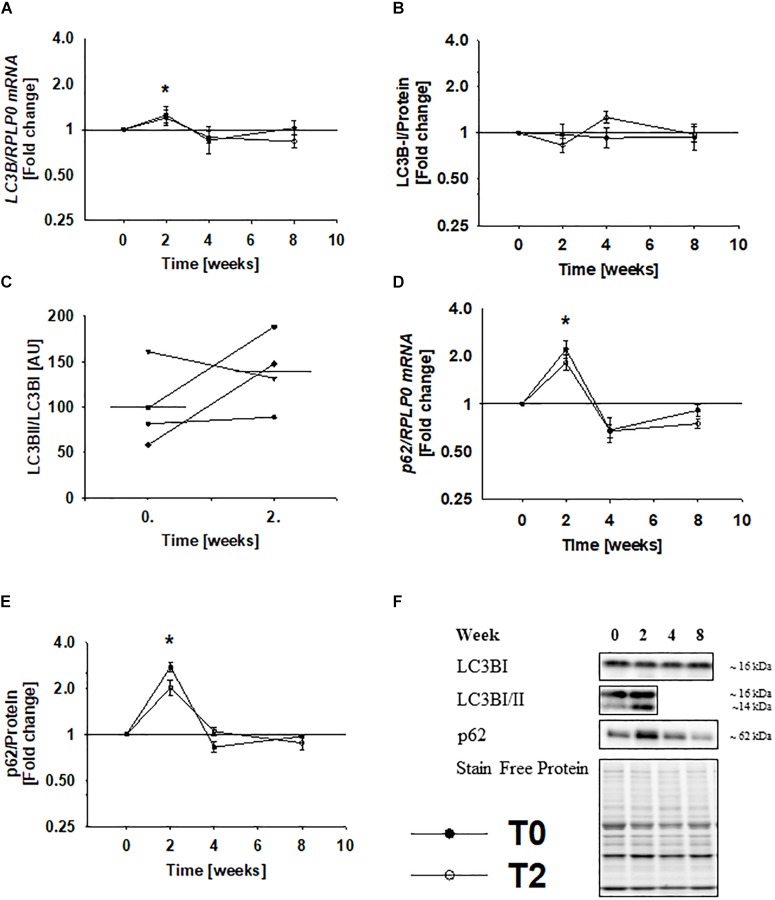
Gene and protein expression of LC3B and p62 increases during immobilization and returns to baseline levels during rehabilitation. mRNA expression of LC3B increased during immobilization and returned to baseline values during rehabilitation **(A)**, while protein expression of LC3B remained unchanged during immobilization and rehabilitation **(B)**. After initial problems with detection of LC3B-II, we extracted proteins from the remaining biopsy material using a lysis buffer containing stronger detergents. This allowed us to examine LC3B lipidation before and after immobilization in four subjects, and LC3B lipidation increased in three of those subjects during immobilization **(C)**. Protein and mRNA expression of p62 increased during immobilization (and decreased to baseline values during rehabilitation **(D,E)**. Values are geometric means ± back-transformed SE. ^∗^*Post hoc* test showed significant change during immobilization. Representative western blots **(F)**. Based on the applied molecular standards, approximated molecular weights are indicated on the right. Filled symbols represent subjects from T0 and open symbols represent subjects from T2.

mRNA expression of LC3B increased by ∼25% during immobilization and returned to baseline levels during 2 weeks rehabilitation (Figure 4A), while protein expression of LC3BI remained unchanged (Figure 4B). This could indicate that autophagy is activated during immobilization and suppressed during rehabilitation, as increased LC3BI production may be accompanied by increased LC3BI removal/lipidation. LC3BI and LC3BII can be separated by western blot based on migration length. However, we were not able to detect LC3BII, possibly due to the low amount of detergents in the applied lysis buffer. Therefore, we extracted proteins from the remaining biopsy material using a lysis buffer containing a higher amount of detergents. This allowed us to examine LC3B lipidation during immobilization in biopsies from four subjects. The ratio of LC3BII to LC3BI increased during immobilization in three of those subjects (Figure 4C), and separate analysis of LC3BI and II demonstrated that this was due to increased LC3BII expression. To examine autophagic status further, we analyzed LC3B in muscle cross-sections by immunofluorescence, but in agreement with previous experience (Suetta et al., 2012), we were unable to detect LC3B with high reproducibility by this method. Protein and mRNA expression of p62 increased by ∼140% during immobilization (Figure 4D,E), which suggests that the recruitment of cytoplasmic material to the autophagosomes increases. Conversely, p62 protein and mRNA levels were completely restored during 2 weeks rehabilitation (Figure 4D,E). Together, these findings indicate that autophagy is activated during immobilization and that physical rehabilitation leads to complete normalization.

### Immobilization Does Not Change Autophagic Signaling Through ULK1

**FIGURE 5:**
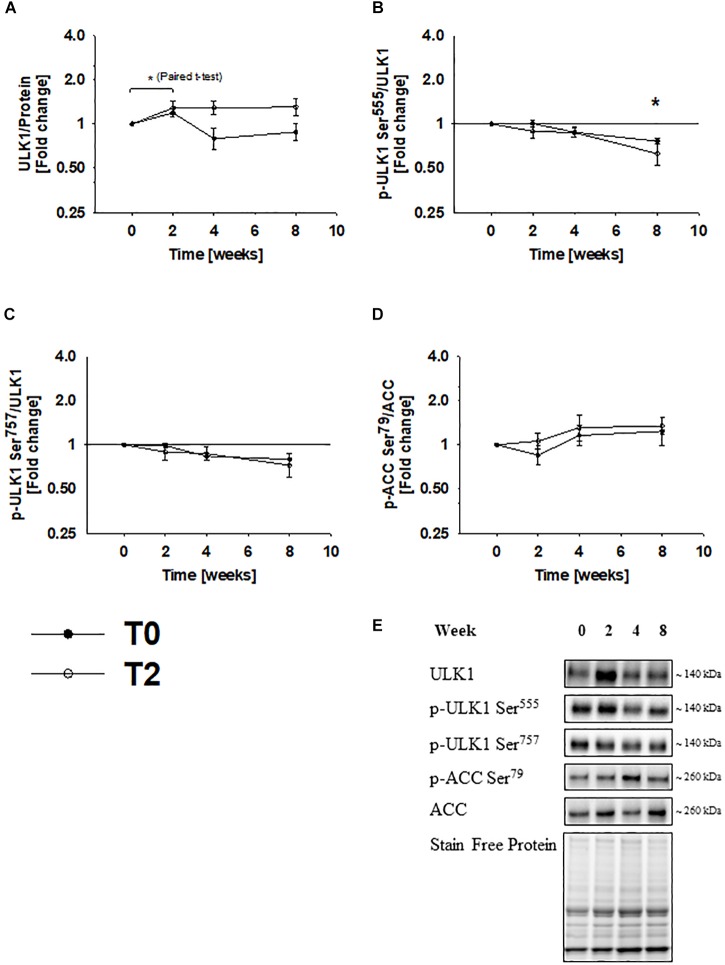
Autophagic signaling trough ULK1 remains unchanged during immobilization and rehabilitation. Based on the mixed linear model with repeated measurements, the change in ULK1 protein expression did not reach statistical significance. However, when a paired *t*-test was used, ULK1 protein expression increased during immobilization and tended decreased toward baseline levels during rehabilitation **(A)**. ULK1 phosphorylation at Ser^555^ and Ser^757^ did not change during immobilization and rehabilitation **(B,C)**. ACC phosphorylation at Ser^79^ did not change throughout the study **(D)**, suggesting that AMPK activity remained unaltered. Values are geometric means ± back-transformed SE. ^∗^*Post hoc* test showed significant change during immobilization. Representative western blots **(E)**. Based on the applied molecular standards, approximated molecular weights are indicated on the right. Filled symbols represent subjects from T0 and open symbols represent subjects from T2.

To address the underlying mechanism responsible for the observed changes in autophagy-related gen and protein expression, we examined signaling through ULK1. Based on the mixed linear model with repeated measurements, the change in ULK1 protein expression did not reach statistical significance. However, when a paired *t*-test was used ULK1 protein expression increased by ∼30% during immobilization and tended (*p* = 0.06) to return to baseline levels during rehabilitation (Figure 5A). ULK1 phosphorylation at Ser^555^ and Ser^757^ did not change during immobilization and rehabilitation (Figure 5B,C). ACC phosphorylation at Ser^79^ did not change during the study, which indicates that AMPKα remained inactive (Figure 5D). Non-transcriptional regulation through ULK1 may therefore not be involved in regulation of autophagy during immobilization and rehabilitation.

### Immobilization Decreases FOXO3a Phosphorylation at Ser^318/321^ and Increases Expression of Autophagy-Related Genes

**FIGURE 6:**
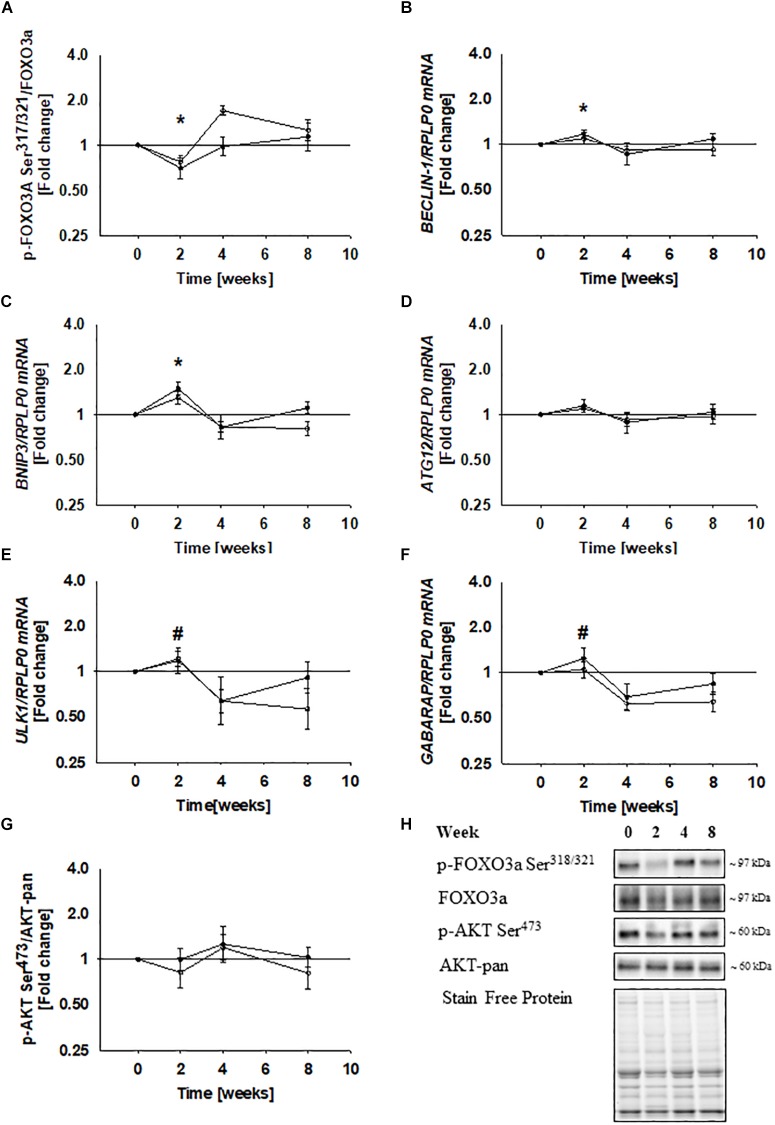
Immobilization decreased FOXO3a phosphorylation at Ser^318/321^ and increases mRNA expression of autophagy-related genes controlled by FOXO3a. FOXO3a phosphorylation at Ser^318/321^ decreased during immobilization and increased to baseline level during rehabilitation **(A)**. mRNA expression of BECLIN-1 and BNIP3 increased during immobilization **(B,C)**, while the increase in ATG12, ULK1, and GABARAPL1 did not reach statistical significance **(D–F)**, although mRNA expression of BECLIN-1, BNIP3, ULK1, and GABARAPL1 decreased during rehabilitation **(B–F)**. mRNA expression of ATG12 remained unchanged during rehabilitation **(D)** AKT phosphorylation at Ser^473^ remained unchanged during the study **(G)**. Values are geometric means ± back-transformed SE. ^∗^*Post hoc* *t*-test showed significant change during immobilization. ^#^*Post hoc* test showed significant change during rehabilitation. Representative western blots **(H)**. Based on the applied molecular standards, approximated molecular weights are indicated on the right. Filled symbols represent subjects from T0 and open symbols represent subjects from T2.

Forkhead box 3a phosphorylation at Ser^318/321^ decreased by ∼25% during immobilization and increased to a level slightly above baseline during rehabilitation (Figure 6A). This indicate that FOXO3a is transcriptionally active during immobilization and inactivated during rehabilitation, as phosphorylation of this site has been shown to be negatively correlated with FOXO3a activity (Tong et al., 2009). This notion fits well with the observed changes in ULK1 protein expression and mRNA expression of LC3B, p62, and MuRF1 (Figure 3C, 4A–D, 5A), as FOXO3a is known to control expression of these genes (Zhao et al., 2007). To examine transcriptional activation of FOXO3a further, we examined mRNA expression of additional five ATG’s under the control of FOXO3a. BECLIN-1 mRNA increased by ∼20% during immobilization and BNIP3 mRNA increased ∼40% during immobilization (Figure 6B,C). BECLIN-1 and BNIP3 mRNA decreased during rehabilitation (Figure 6B,C). The increase in ATG12, ULK1, and GABARAPL1 mRNA during immobilization did not reach statistical significance (Figure 6D–F). However, ULK1, and GABARAPL1 decreased during rehabilitation (Figure 6E,F), suggesting that the expression of these genes indeed was elevated during immobilization, whereas ATG12 mRNA remained unchanged (Figure 6D). AKT phosphorylation at Ser^473^ did not change during the study (Figure 6G). The present study does not allow causal conclusions, but the strong association between FOXO3a phosphorylation and autophagy-related gene expression could imply that FOXO3a regulates autophagy during immobilization and rehabilitation.

## Discussion

In the present study, we demonstrate that autophagy-related gene and protein expression increase during 2 weeks of immobilization and return to baseline within 2 weeks of rehabilitation in human skeletal muscle. Furthermore, our data suggest that FOXO3a is involved in transcriptional regulation these genes, as FOXO3a phosphorylation at Ser^318/321^ decreased during immobilization and returned to baseline during rehabilitation. These data suggests that autophagy is involved in regulation of skeletal muscle mass in humans.

Cross sectional area of the knee muscle-extensors decreased by ∼10% during 2 weeks immobilization and was regained during 2 weeks of physical rehabilitation, which is in agreement with previous observations in humans (Suetta et al., 2012; Wall et al., 2014). The results of the present paper extend these findings by demonstrating that autophagy-related gene and protein expression are regulated in a pattern that mirror muscle mass. In addition to this, we did not observe any changes in MuRF1 and ATROGIN-1 protein expression, which could indicate that the involvement of this system in regulation of muscle mass is limited. This is in substantial contrast to the contribution of this system in rodents where genetic deletion of genes encoding Murf1 and Atrogin-1 protects from disuse-induced muscle loss (Bodine et al., 2001a). However, we did observe increased mRNA expression of MuRF1 during immobilization, but the biological relevance of this is unclear, as it did not translate into altered proteins levels. In this regard, it should be mentioned that gene and protein expression doesn’t always correlated, as several regulatory processes occur after mRNA is made (Vogel and Marcotte, 2012). Although we cannot exclude that the ubiquitin-proteasome system plays a role, these findings indicate that the autophagy-lysosome system plays a more prominent role in regulation of muscle mass in humans.

The ratio of LC3BII to LC3BI increased in three of four subjects during immobilization, demonstrating that the number of autophagosomes is elevated during immobilization. This could reflect impaired fusion with the lysosome and consequent accumulation of autophagosomes. However, increased gene expression of LC3B and stable LC3BI protein levels suggest that LC3B production is stimulated in order to balance an increased turnover of the protein. In line with this, gene and protein expression of p62 increased during immobilization, which could indicate that the recruitment of cytosolic content to the lysosomes is elevated. Few studies have investigated autophagy in human skeletal muscle, but LC3BII and p62 protein expression have been shown to increase during other catabolic conditions, such as prolonged fasting (Vendelbo et al., 2014). Whether autophagy is activated to cover energy demands or to maintain cellular homeostasis remains unknown, but data from the present paper raises the possibility that autophagy is an active catabolic player involved in regulation of muscle mass. This said, we would like to emphasize that no method exists to assess autophagy flux in humans *in vivo*. We have determined the expression of selected autophagy-related genes and proteins and these static measures does not necessarily correlate with dynamic measures of autophagy flux (Jiang and Mizushima, 2015). We have not been able to analyze autophagosome abundance using electron microscopy, as no tissue was collected for this type of analysis. It should also be mentioned that some autophagy-related proteins accumulate in conditions with defective autophagy, including myositis, and sarcopenia (Nogalska et al., 2009; Sakuma et al., 2016). For example, the induction of autophagy in response to disuse is impaired in skeletal muscle from aged mice (O’Leary et al., 2013), and this is associated with accumulation of p62 protein (O’Leary et al., 2013; Sakuma et al., 2016). Denervation has indeed been shown to suppress autophagy in mice (Quy et al., 2013). Thus, we cannot exclude that accumulated p62 levels in the present study reflects that muscle atrophy is associated with impaired autophagy. If this concept is applicable, autophagy doesn’t seem to be activated in atrophied muscle after immobilization. Our study does not allow us to make strong conclusions on this issue, but future studies should focus on developing methods to assess autophagy flux in humans *in vivo*.

Signaling through ULK1 is activated during exercise and fasting in human skeletal muscle (Vendelbo et al., 2014; Moller et al., 2015; Moller et al., 2018). However, ULK1 phosphorylation at Ser^555^ and Ser^757^ did not change in the present study, which indicate that non-transcriptional regulation is not responsible for autophagic regulation during immobilization and rehabilitation. Thus, autophagy must be regulated by distinct mechanisms when involved in fasting and exercise adaptation compared to regulation of muscle mass. FOXO3a phosphorylation at Ser^318/321^ decreased during immobilization, which is consistent with transcriptional activation (Huang and Tindall, 2007). Members of the Foxo family are known to control the expression of autophagy-related genes, including ULK1 and p62 (Milan et al., 2015), and might therefore explain the increased expression of these proteins. To investigate transcriptional activity of FOXO3 more thoroughly, we analyzed mRNA expression of seven autophagy-related genes controlled by FOXO3a. Four of these were upregulated and further three tended to increase. We cannot make strong mechanistic conclusions based on the present study, but these findings could imply that FOXO3a is responsible for transcriptional activation of autophagy during immobilization in human skeletal muscle. Increased mRNA expression of MuRF1 during immobilization further supports that FOXO3a was activated, as gene expression of MuRF1 is tightly controlled by FOXO’s (Milan et al., 2015). DNA binding activity assays could have provided further evidence to support this association, but as mentioned previously no biopsy material is left from the study. Although AKT represents a potential upstream kinase for FOXO3a, the changes in AKT phosphorylation at Ser^473^ did not reach statistical significance. However, AKT phosphorylation has previously been shown to decrease during a similar immobilization protocol in human skeletal muscle (Sakuma et al., 2009). Thus, accumulating evidence suggest that AKT/FOXO signaling could be involved during muscle atrophy in humans.

Studies using tracer techniques report that suppressed protein synthesis contributes to muscle wasting in humans (de Boer et al., 2007; Glover et al., 2008), but the molecular events underlying this remain unclear, as the components controlling translation initiation and elongation appear unchanged (de Boer et al., 2007). As presented previously, mTOR phosphorylation at Ser^2448^ did not change during immobilization or rehabilitation in the present study, and we did not find any changes in phosphorylation of the mTORC1 substrates 4EBP1 Thr^37/46^ and p70S6K Thr^389^ either. Thus, we do not find molecular support to suggest, that protein synthesis decreases during immobilization by suppression of mTORC1 in human skeletal muscle. The negative protein balance observed during immobilization might therefore be a consequence of alternative pathways that suppress protein synthesis.

After immobilization, subjects entered 6 weeks of rehabilitation consisting of progressive resistance training. Subjects were divided into two groups: one group consumed a protein rich meal immediately after each training session, and the other group consumed a similar meal 2 h after each training session. This was done to determine whether or not timing of protein intake influences the rate of muscle regain. As previously described, there was no difference in the rate muscle regain during rehabilitation (Larsen et al., 2001), and in accordance to this, we did not observe any differences between the groups on the molecular targets investigated in the present paper. Thus, timing of exogenous amino acids does not seem to affect the molecular mechanisms that govern the rate of muscle regain after immobilization.

In conclusion, immobilization of a lower limb decreases muscle mass and increases autophagy-related gene and protein expression in human skeletal muscle. Activation of FOXO3a is likely to serve as the underlying transcriptional regulator, as FOXO3a phosphorylation decreases during immobilization. Restoration of muscle mass by 2 weeks rehabilitation completely normalizes autophagy-related gene and protein expression as well as FOXO3a phosphorylation. These data suggests, to our knowledge for the first time, that FOXO3a-dependent regulation of autophagy is involved in the regulation of muscle mass in humans.

## Ethics Statement

The study was approved by the Ethics Committee of Copenhagen (j.no.H-1-2010-016). All participants gave informed written consent to participate in the study. Consent for publication has been obtained from the participants.

## Author Contributions

AM, MV, CC, PS, MK, NM, MH, and NJ conceived and designed the research. AM, CC, PS, and MH performed the experiments. AM, PS, and NJ analyzed the data. AM, MV, PS, MK, MH, and NJ interpreted the results of the experiments. AM prepared the figures and drafted the manuscript. AM, MV, CC, PS, MK, NM, MH, and NJ approved the final version of the manuscript.

## Conflict of Interest Statement

The authors declare that the research was conducted in the absence of any commercial or financial relationships that could be construed as a potential conflict of interest.

## Abbreviations

4EBP1: eIF4E binding protein 1
AMPKα: AMP-activated protein kinase α
ATG: autophagy-related protein
ATROGIN-1: F-box only protein 32
BNIP3: BCL2/adenovirus E1 B 19 kDa interacting protein 3
CSA: cross sectional area
FOXO3a: Forkhead box 3a
eIF4E: Eukaryotic initiation factor 4E
GABARAP: Gamma-aminobutyric acid receptor-associated protein
LC3B: microtubule-associated protein 1 light chain 3
mTORC1: mammalian target of rapamycin complex 1
MuRF1: muscle-specific ring finger protein 1
p62: sequestosome-1
p70S6K: p70S6 kinase 1
PI3K: phosphatidylinositol 3-kinase
